# The Relationship between Pre-Anesthetic Analgesia and Nociception (ANI) and Propofol Injection Pain among Patients Receiving Remifentanil: A Prospective, Randomized, Controlled Study

**DOI:** 10.3390/medicina60020273

**Published:** 2024-02-05

**Authors:** Cheolhyeong Lee, Cheol Lee, Junsung Lim, Jeongki Park, Jaehak Jung, Hayoung Lee, Myeongjong Lee

**Affiliations:** 1 Department of Anesthesiology and Pain Medicine, Wonkwang University School of Medicine Hospital, 895 Muwang-ro, Iksan 54538, Republic of Korea; leecheolhyeong@gmail.com (C.L.); sy4957@naver.com (J.L.); lyrist1@naver.com (J.P.); 2 Department of Obstetrics and Gynecology, Wonkwang University School of Medicine Hospital, 895 Muwang-ro, Iksan 54538, Republic of Korea; 3 Department of Nursing, Wonkwang University School of Medicine Hospital, 895 Muwang-ro, Iksan 54538, Republic of Korea; julia7071@naver.com; 4 Department of Anesthesiology and Pain Medicine, Konkuk University Medical School, 82 Gugwondae-ro, Chungju 27376, Republic of Korea; gooddr21@kku.ac.kr

**Keywords:** analgesia, anesthesia, nociception, pain, propofol, remifentanil

## Abstract

*Background and Objectives*: The analgesia/nociception index (ANI) potentially monitors nociceptive status during anesthesia, but its link to preoperative pain sensitivity is unclear. We investigated the relationship between pre-anesthetic ANI scores and propofol injection pain (PIP) in patients receiving remifentanil. *Materials and Methods*: This study included 124 male patients aged 19–60 undergoing general anesthesia (ASA class I or II). Patients were randomized to group R (*n* = 62, remifentanil 4 ng/mL) or group C (*n* = 62, saline). The primary outcome was the association between PIP and ANI. Secondary outcomes included the incidence and severity of PIP or rocuronium-induced withdrawal movement (RIWM) and their association with ANI. *Results*: PIP and RIWM incidence and severity were lower in group R than in group C. A weak negative correlation between PIP and ANI at pre-induction *(r_pb_* = −0.21, *p* = 0.02, *r_pb_* = −0.37, *p* < 0.01) and a moderate negative correlation during propofol injection (*r_pb_* = −0.48, *p* = 0.02) were observed. A significant negative correlation was found between RIWM and ANI during rocuronium injection (τb = −0.61, *p* < 0.01). AUC, cut-off value, specificity, and sensitivity in ANI at pre-induction for predicting PIP were 0.67 (*p* = 0.02), 59, 76%, and 55%, respectively. AUC, cut-off value, specificity, and sensitivity in ANI during propofol injection for PIP were 0.77 (*p* < 0.01), 65, 81%, and 67%, respectively. *Conclusions*: ANI scores demonstrated significant differences between groups, suggesting potential predictive value for PIP despite the low pre-induction AUC value. This study highlights the potential of using ANI scores to predict and manage PIP in patients receiving remifentanil.

## 1. Introduction

Due to its rapid onset, short duration, easy titration, and favorable side effect profile, propofol is widely used for inducing anesthesia. However, intravenous injection causes pain for approximately 60% of patients, with a significant proportion reporting severe or excruciating pain [1]. To alleviate this pain, researchers have explored the use of remifentanil, an opioid analgesic, as a pretreatment before propofol injection. The previous studies reveal that various effect–site concentrations (Ce) of remifentanil have been used to prevent propofol injection pain (PIP) [2,3,4,5]. The optimal Ce may vary depending on the study design, patient characteristics, and anesthesia protocol. Therefore, an anesthesiologist should determine the appropriate remifentanil Ce based on individual patient needs response and safety considerations.

In the field of anesthesiology, extensive research has been dedicated to exploring the relationship between a patient’s inherent pain perception prior to surgery and the level of postoperative pain experienced. These investigations, incorporating methods like quantitative sensory testing, have demonstrated that preoperative pain assessments can potentially predict between 4% and 54% of the variance in postoperative pain experiences. This wide range of predictability is attributed to differences in testing paradigms and stimulation techniques used in these studies [6,7,8,9].

Effective monitoring of nociception, the neural process of encoding and processing noxious stimuli, is crucial not only for reducing acute pain but also for advancing toward a more automated, nuanced approach to anesthesia and analgesia management [10,11]. A notable tool in this regard is the Analgesia Nociception Index (ANI), developed by Mdoloris Medical Systems. The ANI offers a dimensionless score ranging from 0 to 100, which is calculated based on the analysis of the high-frequency component of heart rate variability, representing a physiological response to pain. Scores on the higher end of this scale, particularly between 90 and 100, indicate effective suppression of pain, suggesting that the administration of additional analgesics may not be necessary. On the other hand, lower ANI scores, especially those between 0 and 49, signal inadequate pain control, indicating a need for prompt intervention with additional pain management strategies. This tool underscores the importance of individualized patient care in perioperative pain management, providing a quantitative method to assess and respond to a patient’s pain in real time.

The Analgesia Nociception Index (ANI) represents a pivotal advancement in the realm of anesthesiology, particularly for monitoring nociception and pain responses during general anesthesia in adult surgical cohorts. This technology is ingeniously crafted to aid healthcare practitioners in accurately evaluating patients’ pain levels, thereby facilitating the optimization of analgesic delivery. The overarching objectives of incorporating ANI are to mitigate pain experiences, curtail the consumption of opioids, and consequently diminish the incidence of opioid-related adverse outcomes [12,13]. Despite its proven efficacy in sedated patients, the applicability and effectiveness of ANI in conscious patients remain an area shrouded in ambiguity, necessitating more exhaustive research endeavors [14,15,16].

The current investigative study pivots around the hypothesis that the ANI, primarily employed to assess nociceptive states during anesthesia, might exhibit a correlation with an individual’s preoperative pain sensitivity. This research, therefore, delves into the potentiality of pre-anesthetic ANI scores, evaluated both prior to induction and amidst propofol administration, as predictive indicators for post-induction pain (PIP) in patients administered a constant effect–site concentration (Ce) of 4 ng/mL of remifentanil. The focus is to unravel the predictive capacity of ANI concerning postoperative pain manifestations, thereby augmenting the precision of pain management strategies in surgical settings.

## 2. Materials and Methods

### 2.1. Study Design

It was approved by the Institutional Review Board (IRB) of Wonkwang University Hospital in February 2021 that a prospective, randomized, and controlled study (registration no. 2021-02-002) would be conducted. In accordance with the Helsinki Declaration of 2013, this study was conducted at the University Hospital from March through April 2023. Clinicaltrials.gov has registered the study (NCT05049577).

### 2.2. Participants

A total of 124 male patients aged 19–60 years scheduled for general anesthesia with an ASA class I or II were included in this study. Due to altered pain perception during the menstrual cycle, female patients were excluded from the study. Patients receiving beta-blockers, ketamine, clonidine, vasoactive drugs (e.g., metaraminol, ephedrine), neostigmine, atropine, or glycopyrrolate were also excluded. Those with venous access problems in the forearm, allergies to propofol or rocuronium, chronic pain, or who took sedatives or analgesics within 24 h were also excluded.

### 2.3. Randomization and Procedure

A 1:1 allocation and two-block randomization were used to stratify the patients for the randomization in Stata 9.0 (StataCorp, College Station, TX, USA). Simple randomization procedures (computerized random numbers) were used to assign all patients to one of two groups: group R (*n* = 62), in which remifentanil was administered at a Ce of 4 ng/mL before propofol administration, and group C (*n* = 62), in which saline was administered at the same volume as remifentanil. According to Minto’s conceptual model, remifentanil was infused using a target-controlled infusion system (Orchestra^®^ from Fresenius Vial, Brézins, France). Upon arrival of the patient in the operating room, their ANI value was measured, which was deemed as the baseline ANI score. The pre-induction ANI score was measured when the concentration of remifentanil was a Ce of 4 ng/mL. The ANI score was measured during propofol injection and rocuronium injection. The ANI scores of the Group C patients were measured similarly to the Group R patients, considering age, height, and weight as they were receiving saline.

In the present study, we tried to find out the relationship between the ANI score and PIP before the introduction of anesthesia with propofol using Ce 4 ng/mL, which has been suggested to be an appropriate concentration of remifentanil in many studies to reduce PIP [3,4,5].

The allocation of patients to groups was blinded. The anesthetic procedures were all performed by two attending anesthesiologists. Anesthesia induction was performed according to the study protocol by one attending anesthesiologist. All outcomes were measured throughout the perioperative period by the other attending anesthesiologist.

### 2.4. Anesthesia and Perioperative Care

All patients had an intravenous 18-gauge cannula inserted in the forearm by nurses in the morning before surgery. All patients did not receive any premedication. A BIS monitor, electrocardiography, invasive arterial blood pressure measurement, and pulse oximetry were used to assess all patients in the operating room. Inducing anesthesia was accomplished via injection in all patients using 2 mg/kg of 1% propofol (long-chain triglyceride (LCT) emulsion) over 30 s (considering arm-brain circulation time (40 s^−1^ min)) [17], followed by injection of 0.6 mg/kg of 1% rocuronium over 10 s, when the BIS value decreased below 60.

An anesthesiologist noted patient movements during and after propofol and rocuronium administration. Patients were evaluated using numeric rating scales (NRSs), scoring pain intensity from 0 to 10 after receiving half-dose and full-dose propofol injections. The scale extended from ‘0’, signifying no pain (e.g., “no pain”), to ‘10’, indicating extreme pain (e.g., “worst pain imaginable”).

The anesthesiologist assessed patients’ pain levels to determine the severity of PIP. The patient received a 15 s administration of a half-dose of propofol. The attending anesthesiologists asked a question after giving the patient the remaining propofol. They recorded the NRS pain score if the patient was unable to answer verbal questions after receiving the full dose. In case of no response, they administered a half dose and recorded the score again. A higher NRS score was recorded for PIP pain severity, regardless of dose.

The anesthesiologist observed patient movement during and after rocuronium administration. Reactions to rocuronium administration were classified according to this scale: 1 (none)  =  no response; 2 (mild)  =  movement at the wrist only; 3 (moderate)  =  movement involving the upper arm or shoulder; and 4 (severe)  =  movement in more than one extremity or a generalized response.

All patients received an end-tidal concentration of 1 minimum alveolar concentration (MAC) of desflurane, which was adjusted in 1 Vol% increments through titration to maintain mean arterial blood pressure (MAP) within ±30% mmHg, heart rate (HR) within ±30%, and bispectral index (BIS) between 40 and 60. Hypotension and bradycardia were defined as MAP < 60 mmHg and HR < 50 bpm, respectively. In the case of hypotension, 250 mL of lactated Ringer’s solution was administered to the patient. If blood pressure did not improve, the patient was administered 10 mg of ephedrine. On the other hand, 0.5 mg of atropine was administered to patients with bradycardia.

After surgery, neuromuscular blockade was reversed using pyridostigmine 0.2 mg/kg mixed with glycopyrrolate 0.04 mg/kg when the train-of-four ratio (TOF) reached 25%. Patients were extubated when their TOF ratio was >0.9 and they began spontaneous breathing; their BIS values increased to 80. To manage postoperative pain, a PCA pump was utilized. This pump contained 800 µg of fentanyl, 150 mg of ketorolac, and 0.6 mg of ramosetron, which was diluted in 150 mL of saline solution. The pump was programmed to deliver a 2 mL/hour basal infusion rate, along with patient-triggered bolus doses of 0.5 mL. To prevent excessive dosing, a 15 min lockout interval was implemented. Upon arrival at the post-anesthesia care unit, pain intensity was evaluated using a 100 mm linear Visual Analog Scale (VAS). If the VAS score was above 50 mm, 100 µg fentanyl was administered intravenously for pain management. If the VAS score was below 40 mm or upon patient request, a 30 mg ketorolac injection was provided.

### 2.5. Outcome Measures

The primary outcome measure was the association between the incidence of PIP and ANI. Secondary outcome measures included incidence and severity of PIP or RIWM, and the association of RIWM and ANI, mean arterial pressure MAP, HR, and BIS were recorded at various time points (baseline, pre-induction, propofol injection, and rocuronium injection).

### 2.6. Sample Size and Statistical Analysis

The sample size for this study was determined using PASS 2008 software (NCSS, LLC, Kaysville, UT, USA). An initial examination indicated that the percentages of patients experiencing PIP during propofol injection were 43% and 19% for the two groups, respectively. Therefore, a sample size of 56 patients per group was needed to detect a significant difference with 80% power and an α-coefficient of 0.05. After accounting for an estimated 10% dropout rate, the final sample size included 50 patients in each group. Statistical analyses were conducted using SPSS version 18.0 (SPSS Inc., Chicago, IL, USA). Data are reported as mean ± SD, median (interquartile range), or the number (percentage) of patients. To compare the groups, independent *t*-tests or Mann–Whitney U tests were employed for continuous variables based on normality. In contrast, χ^2^ tests or Fisher’s exact tests were utilized for categorical variables as appropriate. Correlations between parameters were analyzed using point-biserial correlation (*r_pb_*) or Kendall’s tau-b correlation (τb) tests.

## 3. Results

**Figure 1:**
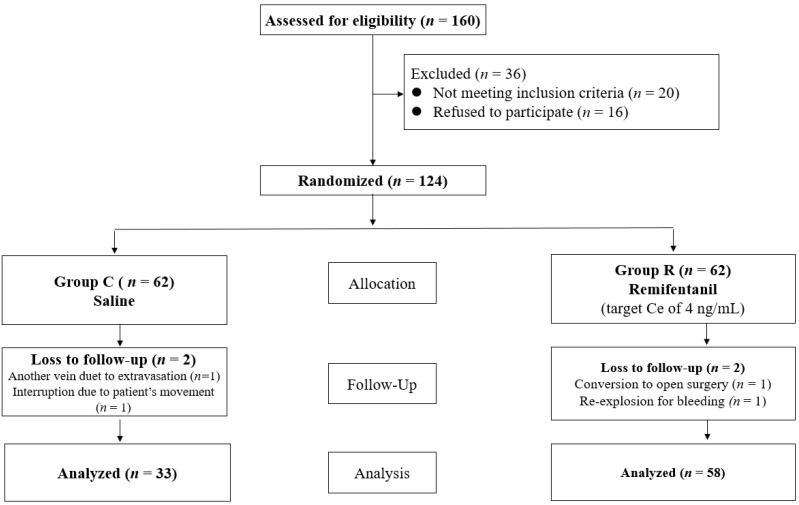
Consort flow diagram.

In the study, 160 patients were rigorously evaluated for eligibility, leading to the exclusion of 36 individuals. Among these, 20 patients failed to meet the predetermined inclusion criteria, while 16 patients opted not to participate. Following the randomization process, 124 patients were administered the specified medication. However, post-enrollment, six patients were withdrawn from the study owing to a range of complications, which included the necessity to utilize an alternative vein due to extravasation, interruptions in the injection process caused by patient movement, and an inability to communicate with the patient as a result of deep sedation (Figure 1).

No significant differences were observed between the two groups concerning age, height, BMI, ASA classification, surgery type, hypotension, bradycardia, desaturation, chest wall rigidity, baseline ANI score, baseline MAP, baseline HR, and baseline BIS (Table 1 and Table 2).

A significant difference was noted between group R and group C with regard to ANI scores at pre-induction, during propofol injection, and during rocuronium injection, as well as MAPs at pre-induction (*p* < 0.01), during propofol injection (*p* < 0.01), and during rocuronium injection (*p* < 0.01), HRs at pre-induction (*p* = 0.02), during propofol injection (*p* < 0.01), and during rocuronium injection (*p* < 0.01), and BISs at pre-induction (*p* < 0.01), during propofol injection (*p* < 0.01), and during rocuronium injection (*p* = 0.012) as well (Table 2). The incidence of PIP, NRS for PIP severity, and the incidence and severity of RIWM were lower in group R than in group C (*p* < 0.01) (Table 3).

PIP and RIWM were not associated with ANI at baseline (*r_pb_* = −0.03, p = 0.78, *r_pb_* = −0.01, *p* = 0.93). A significant weak and negative correlation was observed between the PIP and the ANI at pre-induction (*r_pb_* = −0.21, *p* = 0.02, *r_pb_* = −0.37, *p* < 0.01). PIP significantly and negatively correlated with ANI during propofol injection (*r_pb_* = −0.48, *p* = 0.02). There was a statistically significant negative correlation between RIWM and ANI during rocuronium injection (τb = −0.61, *p* < 0.01) (Table 4).

**Figure 2:**
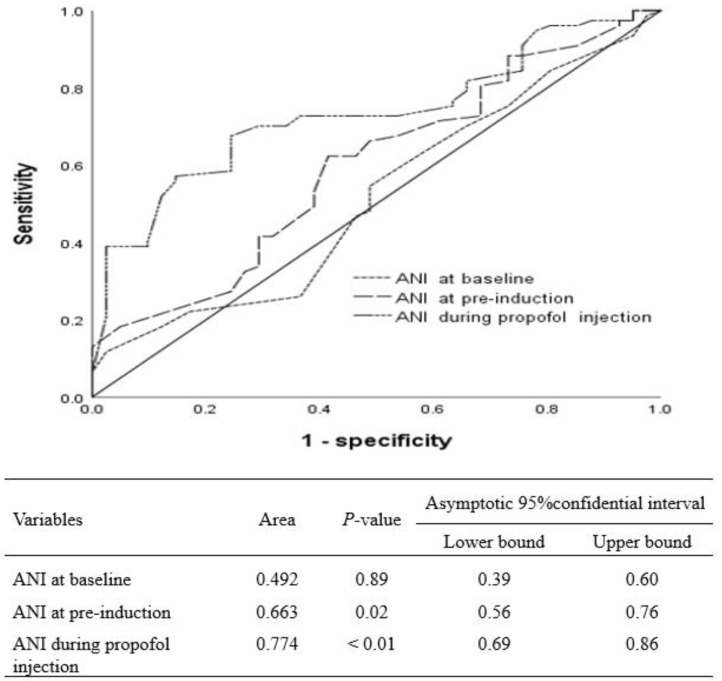
A ROC (receiver operating characteristics) curve and AUC (area under the ROC curve) of ANI (analgesia/nociception index) in predicting propofol injection pain.

The area under the curve (AUC) for the receiver operator characteristics (ROC) curve, cut-off value, specificity, and sensitivity in ANI at pre-induction for predicting PIP were 0.67 (*p* = 0.02, 0.56 < Confidence Interval (CI) < 0.76), 59, 76%, and 55%, respectively. AUC, cut-off value, specificity, and sensitivity in ANI during propofol injection for PIP were 0.77 (*p* < 0.01, 0.69 < CI < 0.86), 65, 81%, and 67%, respectively (Figure 2).

## 4. Discussion

Our current findings indicate that the optimal cut-off values for predicting PIP (NRS > 0) in conscious patients are 59 for ANI at the pre-induction stage and 65 for ANI during propofol injection. These values differ from the manufacturer-recommended cut-off value of 50 for ANI, as they are higher in both cases. The cut-off values identified in this study align with the pain thresholds suggested by previous research on ANI for conscious patients experiencing pain [18]. Different respiratory patterns affect ANI values differently between conscious and anesthetized patients, which may explain the difference in cut-off values between them [19].

These findings are clinically important because they challenge the manufacturer-recommended cut-off value of 50 for ANI by suggesting that the optimal cut-off values for predicting PIP in conscious patients are 59 for ANI at the pre-induction stage and 65 for ANI during propofol injection. These higher cut-off values align with pain thresholds suggested by previous research on ANI for conscious patients experiencing pain [14,18,20]. The identification of more accurate cut-off values for predicting PIP can help anesthesiologists target pain management strategies based on the patients’ pain levels during anesthesia administration. Consequently, patients can receive better care, experience less discomfort during procedures, and potentially recover more quickly. Moreover, these findings contribute to a better understanding of the relationship between ANI scores and pain thresholds, which can be valuable for future research and clinical practice.

In conscious patients, the ANI threshold for distinguishing NRS > 3 in the post-anesthesia care unit was 57 for propofol and remifentanil anesthesia and 59 for sevoflurane-based anesthesia. The cut-off value of ANI for detecting pain (NRS > 0) in both types of anesthesia was 63, regardless of the type used [14,18,20]. It is generally accepted that in order to be beneficial, the accuracy of any diagnostic technique requires an AUC greater than 0.5, and generally speaking, it is considered acceptable if the AUC exceeds 0.8 [21]. The present study showed that the AUC for ANI was 0.67 for predicting PIP pre-induction and 0.77 for discriminating PIP during propofol injection. The low AUC values of these indicators may be a consequence of their poor performance. Previous studies also found that the AUCs of these indicators were below 0.8 in conscious patients except for one (AUC of ANI:0.89) [14,18,22,23]. When the AUC is greater than 0.5, it can be used as a diagnostic test.

Another important result of this study is the significant correlations observed between the incidence of PIP and RIWM. Specifically, PIP had a significant moderate and negative correlation with ANI during propofol injection (*r_pb_* = −0.48, *p* = 0.02) and a significant negative correlation between RIWM and ANI during rocuronium injection (τb = −0.61, *p* < 0.01). These correlations indicate that monitoring ANI scores could be useful for predicting the likelihood of PIP and RIWM during anesthesia administration, potentially allowing for more personalized and effective pain management during these procedures.

In analyzing the limitations of this research, several key factors emerge that necessitate careful consideration. Firstly, the deliberate exclusion of female patients due to the variability in pain perception during menstrual cycles presents a notable limitation. This exclusion significantly restricts the applicability and relevance of the study’s findings to a more inclusive demographic that embodies both sexes. Secondly, this study’s focus on a specific subset of male patients aged between 19 and 60 years, all classified under ASA physical status I or II, potentially hinders the extrapolation of results to broader patient groups. Particularly, it limits insights into the implications for older adults or those with more complex health conditions (higher ASA classifications). Thirdly, there is the issue of the small sample size: this study had a relatively small sample size of 124 participants, with 62 participants in each group. A larger sample size could have provided more robust and reliable results. Fourthly, this study was conducted at a single center, which may have limited the external validity of its findings. The findings may be influenced by specific institutional practices, patient demographics, or healthcare systems, limiting the generalizability to other settings. Fifthly, the potential for confounding variables, despite the implementation of randomization, cannot be overlooked. It is plausible that certain unaccounted or unidentified factors might have inadvertently influenced the study outcomes. Sixthly, this study’s reliance on self-reported pain scales, such as numeric rating scales, introduces an element of subjectivity and variability. This reliance can impact the precision and reliability of pain assessments. Seventhly, there was a reliance on self-reported pain scales: this study used numeric rating scales for pain assessment, which relies on patients’ subjective reporting. This approach may introduce variability and subjectivity in pain assessment, affecting the accuracy and reliability of the results. Finally, despite the fact that this study did not examine the possible effects of different propofol and remifentanil concentrations on the outcomes, this may have implications for the clinical applicability of the findings.

## 5. Conclusions

In conclusion, the pre-anesthetic ANI scores (before anesthesia induction and during propofol administration) were predictive of PIP in patients receiving a target remifentanil concentration of 4 ng/mL. Despite the low pre-induction AUC, the ANI scores exhibited significant differences between the two groups at various time points, suggesting a potential predictive value for PIP. Remifentanil preconditioning effectively reduces PIP. Moreover, ANI has been demonstrated as an effective tool for monitoring nociception during anesthesia induction with propofol, particularly in patients receiving remifentanil. Further research is warranted to validate these findings and explore their practical implications.

## Figures and Tables

**Table 1:** Demographic data.

	Group C (*n* = 60)	Group R (*n* = 58)	*p*-Value
Age (y)	50.8 ± 8.4	50.5 ± 7.2	0.85
Height (cm)	168.8 ± 5.2	169.4 ± 5.1	0.48
Weight (kg)	73.3 ± 7.8	72.9 ± 7.5	0.76
BMI (kg/m^2^)	25.7 ± 2.1	25.4 ± 2.2	0.40
ASA (I/II)	43/17 (71.7/28.3)	42/16 (72.4/27.6)	0.93
Type of surgery			0.95
Spine surgery	19 (31.7)	18 (31.0)	
Laparoscopic gastrointestinal surgery	22 (36.7)	20 (34.5)	
Laparoscopic urologic surgery	19 (31.7)	20 (34.5)	

Data are presented as mean ± standard deviation (SD) or number (%). BMI: body mass index. ASA: American Society of Anesthesiologists physical status.

**Table 2:** ANI, MAP, HR, BIS monitoring and complications during medication administration.

	Group C (*n* = 60)	Group R (*n* = 58)	*p*-Value
ANI score at baseline	54.1 ± 4.8	54.5± 5.9	0.70
ANI score at pre-induction	53.4 ± 5.0	73.0 ± 8.5	<0.01
ANI score during propofol injection	44.9 ± 8.8	68.7 ± 9.7	<0.01
ANI score during rocuronium injection	35.7 ± 8.0	62.9 ± 8.5	<0.01
MAP at baseline (mmHg)	103.7 ± 7.5	104.4 ± 6.7	0.63
MAP at pre-induction (mmHg)	101.6 ± 6.3	94.1 ± 8.3	<0.01
MAP during propofol injection (mmHg)	96.1 ± 6.6	91.3 ± 8.3	<0.01
MAP during rocuronium injection (mmHg)	95.6 ± 7.6	90.9 ± 8.0	<0.01
HR at baseline (beats/min)	65.0 ± 12.3	64.2 ± 14.3	0.75
HR at pre-induction (beats/min)	72.5 ± 12.7	66.9 ±12.0	0.02
HR during propofol injection (beats/min)	73.8 ± 11.3	62.8 ± 11.6	<0.01
HR during rocuronium injection (beats/min)	76.4 ± 12.6	62.1 ± 11.3	<0.01
BIS at baseline	97.2 ± 1.0	97.1 ± 0.9	0.42
BIS at pre-induction	97.2 ± 0.9	90.6 ± 1.2	<0.01
BIS during propofol injection	90.8 ± 3.7	85.3 ± 3.9	<0.01
BIS during rocuronium injection	54.7 ± 3.3	53.7 ± 3.3	0.012
Hypotension	0 (0)	2 (3.4)	0.15
Bradycardia	0 (0)	3 (5.2)	0.07
Desaturation	0 (0)	0 (0)	
Chest wall rigidity	0 (0)	0 (0)	

Data are presented as mean ± standard deviation (SD) or number (%). ANI: Analgesia/nociception index. MAP: mean arterial pressure. HR: heart rate. BIS: bispectral index.

**Table 3:** The incidence and severity of PIP and RIWM.

	Group C (*n* = 60)	Group R (*n* = 58)	*p*-Value
Propofol injection pain	33 (55.0)	2 (3.4)	<0.01
NRS for propofol injection pain severity	3 (0, 3)	0 (0, 0)	<0.01
RIWM			<0.01
Grade 1	4 (6.7)	51 (87.9)	
Grade 2	39 (65.0)	7 (12.1)	
Grade 3	15 (25.0)	0 (0)	
Grade 4	2 (3.3)	0 (0)	

Data are presented as median (Interquartile range) or number (%). PIP: propofol injection pain. RIWM: rocuronium-induced withdrawal movement.

**Table 4:** The correlation of ANI scores with PIP and RIWM.

	Propofol Injection Pain		Rocuronium Withdrawal Movement	
	Point-Biserial Correlation Coefficient *(r_pb_*)	*p*-Value	Kendall’s tau-b	*p*-Value
ANI at baseline	−0.03	0.78	−0.01	0.93
ANI at pre-induction	−0.211	0.02	−0.37	<0.01
ANI during propofol injection	−0.48	<0.01		
ANI during rocuronium injection			−0.61	<0.01

PIP: propofol injection pain. RIWM: rocuronium-induced withdrawal movement.

## Data Availability

The datasets used and/or analyzed during the current study are available from the corresponding author on reasonable request.

## References

[1] Jalota L., Kalira V., George E., Shi Y.Y., Hornuss C., Radke O., Pace N.L., Apfel C.C., Perioperative Clinical Research Core (2011). Prevention of pain on injection of propofol: Systematic review and meta-analysis. BMJ.

[2] Lee J.-Y., Yang H., Choi S.H., Shin D.W., Hong S.-K., Chun D.-H. (2012). The optimal effect-site concentration of remifentanil to attenuate the pain caused by propofol. Korean J. Anesthesiol..

[3] Tak Y.J., Park S.H., Kim S.T. (2009). The effect of pretreatment with two different concentrations of remifentanil on propofol injection pain. Korean J. Anesthesiol..

[4] Chae Y., Min S., Park S., Kim S., Won Y., Cho H. (2011). Reduction of microemulsion propofol-induced injection pain via target-controlled remifentanil infusion. J. Int. Med. Res..

[5] Lee J.-R., Jung C.-W., Lee Y.-H. (2007). Reduction of pain during induction with target-controlled propofol and remifentanil. Br. J. Anaesth..

[6] Werner M.U., Duun P., Kehlet H. (2004). Prediction of postoperative pain by preoperative nociceptive responses to heat stimulation. Anesthesiology.

[7] Aasvang E.K., Hansen J.B., Kehlet H. (2008). Can preoperative electrical nociceptive stimulation predict acute pain after groin herniotomy?. J. Pain.

[8] Hsu Y.-W., Somma J., Hung Y.-C., Tsai P.-S., Yang C.-H., Chen C.-C. (2005). Predicting postoperative pain by preoperative pressure pain assessment. Anesthesiology.

[9] Werner M.U., Mjobo H.N., Nielsen P.R., Rudin A. (2010). Prediction of postoperative pain: A systematic review of predictive experimental pain studies. Anesthesiology.

[10] Ledowski T. (2019). Objective monitoring of nociception: A review of current commercial solutions. Br. J. Anaesth..

[11] Banerjee S., MacDougall D. (2018). Nociception Monitoring for General Anesthesia: A Review of Clinical Effectiveness, Cost-Effectiveness, and Guidelines.

[12] Shahiri T.S., Richebé P., Richard-Lalonde M., Gélinas C. (2022). Description of the validity of the Analgesia Nociception Index (ANI) and Nociception Level Index (NOL) for nociception assessment in anesthetized patients undergoing surgery: A systematized review. J. Clin. Monit. Comput..

[13] Jiao Y., He B., Tong X., Xia R., Zhang C., Shi X. (2019). Intraoperative monitoring of nociception for opioid administration: A meta-analysis of randomized controlled trials. Minerva Anestesiol..

[14] Lee J.-H., Choi B.-M., Jung Y.-R., Lee Y.-H., Bang J.-Y., Noh G.-J. (2020). Evaluation of Surgical Pleth Index and Analgesia Nociception Index as surrogate pain measures in conscious postoperative patients: An observational study. J. Clin. Monit. Comput..

[15] Koprulu A.S., Haspolat A., Gul Y.G., Tanrikulu N. (2020). Can postoperative pain be predicted? New parameter: Analgesia nociception index. Turk. J. Med. Sci..

[16] Baroni D.A., Abreu L.G., Paiva S.M., Costa L.R. (2022). Comparison between Analgesia Nociception Index (ANI) and self-reported measures for diagnosing pain in conscious individuals: A systematic review and meta-analysis. Sci. Rep..

[17] Dinis-Oliveira R.J. (2018). Metabolic Profiles of Propofol and Fospropofol: Clinical and Forensic Interpretative Aspects. Biomed. Res. Int..

[18] Boselli E., Daniela-Ionescu M., Bégou G., Bouvet L., Dabouz R., Magnin C., Allaouchiche B. (2013). Prospective observational study of the non-invasive assessment of immediate postoperative pain using the analgesia/nociception index (ANI). Br. J. Anaesth..

[19] Le Guen M., Jeanne M., Sievert K., Al Moubarik M., Chazot T., Laloe P.A., Dreyfus J.F., Fischler M. (2012). The Analgesia Nociception Index: A pilot study to evaluation of a new pain parameter during labor. Int. J. Obstet. Anesth..

[20] Ledowski T., Tiong W.S., Lee C., Wong B., Fiori T., Parker N. (2013). Analgesia nociception index: Evaluation as a new parameter for acute postoperative pain. Br. J. Anaesth..

[21] Nahm F.S. (2022). Receiver operating characteristic curve: Overview and practical use for clinicians. Korean J. Anesthesiol..

[22] Yan Q., An H., Feng Y. (2017). Pain assessment in conscious healthy volunteers: A crossover study evaluating the analgesia/nociception index. Br. J. Anaesth..

[23] Charier D., Vogler M.-C., Zantour D., Pichot V., Martins-Baltar A., Courbon M., Roche F., Vassal F., Molliex S. (2019). Assessing pain in the postoperative period: Analgesia Nociception IndexTM versus pupillometry. Br. J. Anaesth..

